# Pragmatic Evaluation of a Health System-Based Employee Weight Management Program

**DOI:** 10.3390/ijerph18115901

**Published:** 2021-05-31

**Authors:** Meghan M. JaKa, Jennifer M. Dinh, Rachael L. Rivard, Stephen D. Herrmann, Joel Spoonheim, Nicolaas P. Pronk, Jeanette Y. Ziegenfuss

**Affiliations:** 1 HealthPartners Institute, Bloomington, MN 55425, USA; Meghan.M.Jaka@healthpartners.com (M.M.J.); Rachael.L.Rivard@healthpartners.com (R.L.R.); Nico.P.Pronk@healthpartners.com (N.P.P.); Jeanette.Y.Ziegenfuss@healthpartners.com (J.Y.Z.); 2 Sanford Research, Sioux Falls, SD 57104, USA; stephen.herrmann@profileplan.com; 3 Sanford Health, Sioux Falls, SD 57117, USA; 4 HealthPartners, Bloomington, MN 55425, USA; Joel.B.Spoonheim@healthpartners.com

**Keywords:** population health, health care, health coaching, worksite wellness, weight loss, obesity prevention

## Abstract

*Objective*: We aimed to evaluate the fidelity and estimate the effectiveness of a novel health system employee weight-management program. *Methods:* Employees participating in a weight loss program consisting of self-monitoring, health coaching and meal replacements optionally enrolled in the 12-month study. Longitudinal, single-arm analyses were conducted evaluating change over time via survey, claims and programmatic data. Token participation incentives were offered for survey completion. *Results*: In total, 140 participants enrolled (51.2 ± 9.8 years; BMI = 33.2 ± 6.5 kg/m^2^; 89.3% female). During 1 year, participants attended 18.0 ± 12.2 coaching appointments and self-reported significant improvements in weight (−8.2 ± 10.5% body weight), BMI (−3.9 ± 6.5 kg/m^2^), fruit/vegetable intake, home food preparation, added sugar, sugar sweetened beverages and life satisfaction (all *p* < 0.05). No significant changes were reported in physical activity, weight-related social support, self-efficacy or healthcare utilization (all *p* > 0.05). *Conclusions*: The findings from this evaluation establish implementation fidelity. Clinically significant self-reported weight loss, coupled with improvements in many weight-related behaviors, suggest the program is an effective weight management tool when offered as an employee well-being program.

## 1. Introduction

Overweight and obesity are associated with increased morbidity and mortality, which in turn have economic impacts related to medical care, productivity and costs in human capital [1]. In the workplace, obesity has widely-documented impacts on well-being and role functioning; employees with obesity have 20% more doctor visits, incur $644 more (U.S. dollars) in medical care costs and are absent from work 3.7 more days per year than those without overweight or obesity [2,3]. Although obesity is multifaceted and particularly challenging to address in the workplace, some promising strategies exist including health coaching [4], structured meal planning including meal replacement [5] and other lifestyle interventions [6].

To help address the impacts of overweight and obesity, the Institute of Medicine’s Committee on Accelerating Progress in Obesity Prevention recommends “Expand[ing] the role of health care providers, insurers, and employers in obesity prevention” [7]. Although many health plans offer commercially available weight loss programs, to our knowledge, few evidence-based employee weight-loss programs have been developed and implemented in close coordination with the healthcare delivery system and insurers [8]. This type of collaboration has the potential not only to improve program design and credibility but also to ensure that programs are affordable for employees. Further, these collaborations allow for a more rigorous evaluation of the impacts of weight loss programs on healthcare-related outcomes (e.g., chronic disease diagnoses, medication and healthcare utilization) via available data sources such as insurance claims.

To date, obesity-related worksite wellness programs have been shown to have a modest effect on weight reduction and healthy weight-related behaviors [9]. However, the rigor with which these programs have been evaluated varies widely or, in some cases, is lacking completely [10,11]. This limits the ability to understand which parts of these programs are or are not effective and inhibits evaluator’s ability to improve future efforts. Using best-practice guidelines for research and evaluation (e.g., TREND or STROBE) [12,13] alongside pragmatic evaluation designs maximizes learnings in an efficient, affordable way. Lastly, to ensure sustainability within the workplace, research is needed to better understand the effect of worksite wellness programs on work-related outcomes such as absenteeism and productivity [9]. Much of the published literature on the effectiveness of weight-loss programs outside the workplace focuses on weight-related behavioral mechanisms, behaviors and outcomes without concurrently measuring impacts on healthcare and worksite outcomes. Combining these with a rigorous assessment of the level to which the intervention was successfully implemented as designed (or “intervention fidelity”) [14] will lead to identification of opportunities to improve programs and increase insurer confidence in offering quality programs to their employer groups and health plan members.

Profile by Sanford (“Profile”, https://www.profileplan.com/, accessed on 1 May 2021) is a behavioral weight management program designed and implemented by a large Midwest integrated health system (Sanford Health). It is a theory-informed, employee-centered program designed to align diet, activity and lifestyle behaviors with weight management through in-person and phone coaching paired with meal replacement offerings and self-monitoring behaviors. The program, founded in behavioral health theory [15,16,17,18,19], was developed by physicians and scientists at Sanford Health following best-practice recommendations from several public health and nutrition organizations [20,21,22,23,24]. Profile utilizes a number of unique strategies such as smart scales, one-on-one coaching and meal planning to improve long-term success. Research has demonstrated that meal replacements are a highly effective tool for short- and long-term weight management when used within a comprehensive behavioral weight management program [5]. Best-practice, evidence-based weight management programs use meal replacements as a tool to simplify behavior change, improve nutritional quality, improve fruit/vegetable intake and manage calorie intake while providing better short- and long-term weight loss than programs using traditional reduced calorie or whole food meal plans.

The program had yet to be tested in a healthcare worksite setting; it is not known how Profile may impact healthcare utilization, workplace productivity, job satisfaction or overall well-being. This pragmatic evaluation was designed to test the impact of Profile on: (1) employee-centered outcomes (weight, weight-related behaviors, health and well-being); and (2) health system- and employer-related outcomes (healthcare utilization, absenteeism and job satisfaction) and to investigate program implementation fidelity including participant satisfaction. In the context of continued increases in obesity across the country [25], a single-arm longitudinal design is an appropriate pragmatic approach to estimate program effectiveness because secular trends suggest weight gain would likely occur in this population over time without intervention. As such, the results of this evaluation will be used to make program improvements, guide future research and inform health system decisions about program offerings for employees and employer groups.

## 2. Materials and Methods

### 2.1. Population and Setting

This evaluation was conducted in an integrated health system (HealthPartners) (one that operates as both a care delivery system and a health insurer) distinct from the organization that developed and owns Profile. This member-governed not-for-profit health system, independent from Sanford Health and Profile, employs more than 26,000 people and serves over 2 million patients and members across the Midwest. The health system has an employer-centered Worksite Health & Population Well-being department housed within a larger health and care engagement division. This department currently offers a range of well-being products to its own employer group as well as to other outside employers and maintains a robust digital platform to provide annual health assessments and health promotion programs. These services are implemented in collaboration with health plan employers and customers at their sites for their employees and dependents. Completion of the health assessment and a qualifying program during the plan year allows participating employees to receive a financial incentive of reduced insurance costs and/or co-pays in the subsequent year, typically totaling $250 U.S. dollars. Within the Worksite Health & Population Well-being department and in partnership with the health system’s human resources department, a team of health program specialists are assigned to the health system’s employer group and offer tailored well-being programming for employees. The programming is a blend of on-site, digital and telephonic activities that support a comprehensive range of health and well-being needs. Activity options are personalized based on prioritized health needs identified through a health assessment and claims data.

Profile is currently offered to all interested health system employees and adult dependents who receive health insurance through their employer. Employees are offered the program through onsite wellness fairs, digital well-being portal promotions, email communications and other employee newsletters. To enroll, interested individuals complete a screening and intake visit in person or via phone. Employees are eligible for the program if, after being screened by Profile coaches, they meet health criteria, do not have certain food allergies and are not receiving certain medical treatment regimens (such as active cancer treatment). Participants who complete at least 20 Profile coaching sessions during the year are eligible for the insurance incentive. The incentive was not tied to program outcomes (e.g., reported weight loss or behavior change), rather to participation in the program.

### 2.2. Eligibility Criteria

Inclusion criteria were: (1) employed by the named health system above or adult dependent covered by the health system’s insurance and (2) enrolled in the Profile program between March 2018 and June 2019. Exclusion criteria included: (1) age younger than 18 or older than 64; (2) self-reported being pregnant or planning to become pregnant at the time of enrollment; and (3) participating in another weight loss or lifestyle management program. Profile program participants were eligible to enroll in the study regardless of their participation in any of the program components described below.

### 2.3. Profile Program

Profile is a personalized weight loss and weight-loss maintenance program that utilizes a one-on-one lifestyle behavior coaching program focused on healthy diet and physical activity. The design of Profile is informed by behavior change theory including Self Determination Theory [17,18] and Social Cognitive Theory [15,16]. Program members receive a Wi-Fi Smart scale to bring home that syncs with an interactive web platform and coaching application. Profile recommends a balanced, reduced-calorie meal plan using a combination of grocery foods and meal replacements to ensure a nutritionally complete diet that includes adequate intake of vitamins and minerals. Programs utilizing meal replacements, such as protein shakes and bars, in combination with grocery foods and coaching have been shown to be superior to traditional reduced calorie meal plans [5,26,27,28,29]. Meal replacements are used as a tool to help simplify food choices during initial behavior change which can reduce cognitive load and increase the chances of success. While ensuring nutritional and caloric goals are met, meal replacements simplify meal planning, food choices and cooking, allowing the Profile member and Profile coach more time to work through the behavioral components necessary to make long-lasting lifestyle changes. The program foods are not a required component of the program but are highly encouraged by program coaches as an effective tool for weight management. In addition to Profile foods, members are expected to purchase additional items from the grocery store to round out their nutrition plan (e.g., lean protein, 4 or more cups of vegetables per day, etc.) Meal replacements are phased out as participants improve nutrition, activity and behavioral knowledge and related skills.

Behavior change and lifestyle modification are promoted through education and one-on-one coaching appointments with a certified Profile coach. Members have access to 30-min weekly coaching appointments for the duration of the program (up to 52 weekly appointments over the course of a one-year membership) and the ability to meet with a single coach or use different coaches. At coaching appointments, coaches and members review conversations, topics and goals from the previous week; discuss new education and behavior topics such as self-compassion and nutrition label reading; and update or set new goals for the upcoming week. Coaching appointments cover the program detail outlined here and focus on health behavior change strategies such as self-monitoring of behaviors and outcomes, goal setting and problem solving. All Profile coaches have at least a bachelor’s degree in a health-related field and receive extensive Profile training including shadowing and practice coaching sessions. All coaches must successfully complete certification which consists of onboarding and training of at least 40 h in their home location and via the Profile learning management system prior to a 3-day in-person Coach Certification course. Following successful completion of training and upon passing the certification exam, Profile coaches are on a 90-day monitoring plan where their consultations are shadowed and their plans and coaching notes are audited. Shadowing and audits continue throughout every coach’s employment. Lastly, all Profile coaches are required to complete 15 credit-hours of continuing education each year to maintain their certification to coach members.

Coaches utilize meal plan protocols to meet dietary needs and personalize to fit individual members, show members how to safely increase their daily activity level and work with members on creating a healthier lifestyle with behavior and habit changes. Coaching is available by phone, video and in-person at Profile locations. Members may also email coaches or send messages over the mobile/web coach app. The program is individually tailored, but typically focuses initially on structured meal plans and meal replacement. Over time, this transitions to fewer meal replacements and more grocery foods as participants acquire and reinforce new knowledge, skills and behaviors to support these changes.

### 2.4. Study Design

This evaluation was designed to help health system leadership assess the effectiveness of Profile when offered as a worksite and health plan incentive program. To allow for adequate time to assess results prior to making subsequent vendor decision and in alignment with the annual Profile program membership, the study was designed to last 12 months. The study was designed by the implementation health system’s affiliated research and evaluation institute (“institute”), in collaboration with leaders from both health systems. The evaluation was also funded in part by both health systems, allowing for alignment of evaluation efforts with strategic goals for the health systems and Profile. However, to maintain appropriate employee confidentiality and minimize potential for perceived bias or coercion, the study was conducted solely by the Institute, which only shared summarized, de-identified results with either organization.

This was a prospective, longitudinal study describing the outcomes associated with individuals who enrolled in Profile up to one year post enrollment. Self-reported outcome data were collected at baseline (immediately following program enrollment) and at 3, 6 and 12 months following enrollment. For parsimony, outcomes are presented for baseline, 3 months and 12 months only as findings at 6 months were not unique. With informed consent, retrospective insurance claims data were collected for one year prior to program enrollment and for the prospective year following enrollment. The study is a longitudinal pre/post design with no randomization or control group. Participants enrolled in the Profile program were responsible for the cost of the program ($99 per year membership) and food (cost varies per member, but it averages $80 per week or $2.34 per meal). The project was reviewed as human subjects research by the HealthPartners Institutional Review Board (IRB) and approved on 2 March 2018 (#A18-037).

### 2.5. Recruitment and Enrollment

All eligible employees who enrolled in the Profile program during the study period were notified about the study through a study recruitment email from a Profile coordinator. The message may have also been reinforced by their Profile coach. The study opportunity was also included in the health system’s employee communications, announcing the Profile program as well as on the institute’s website. Profile enrollees were asked to complete a study interest form. Individuals who completed the interest form were then contacted by research staff to screen for eligibility and assess interest in the study. Eligible and interested individuals discussed the consent form over the phone and were asked to verbally provide informed consent to the study. Later, this was streamlined by offering an online consent process, with a phone number available should interested individuals have questions. To minimize the potential of coercion or undue influence, participants were informed that their participation would be confidential and that participation would not impact their relationship with their employer or health plan or their relationship with Profile and individual responses would not be shared. A copy of the consent form was also emailed to all participants who were enrolled in the study.

### 2.6. Data Collection and Outcome Measures

For the purposes of this study, three data sources were triangulated: study-generated survey data, health system insurance claims data and Profile programmatic data. Each data source and the measures associated are described below.

### 2.7. Survey Methods and Measures

Once individuals consented into the study, they were emailed a 34-item baseline survey to complete online (see Supplementary Materials for the baseline survey). Initial non-responders were sent up to two email reminders over one week and called up to five times over about two weeks, with voicemail reminders left at the first and fifth call attempt. Individuals who did not complete the baseline survey were considered lost to follow-up and were not enrolled in the study or pursued for later survey data collection. After survey completion, they were mailed a $15 Target or Profile gift card (depending on preference) as a thank you for survey completion. Individuals who completed the baseline survey were invited to complete surveys at 3 and 12 months post-enrollment with the same reminder processes and gift card options. Those who completed the 3-month survey were sent a $15 gift card, while 12-month survey completers received a $20 gift card. Survey constructs needed to address research questions were identified in partnership with stakeholders. Where available and described below, existing questions and scales were used or adapted (*n* = 20). For the limited constructs (*n* = 8) where existing questions or scales were not available, questions and response options were designed using best practice [30]. The complete survey was assessed for face validity by pilot testing with stakeholders from both Profile and the health plan and was reviewed for ease of completion with professional telephone interviewers. Survey items asked across time points were asked identically to ensure comparability.

#### 2.7.1. Weight-Related Behaviors and Behavioral Mechanisms

Two items with Likert-scale response options asked participants about binge eating and general social support. Two items asked about prior weight loss attempts and ideal weight loss. Respondents were asked to self-report their height and weight on the survey which were used to calculate body mass index (BMI) and weight status according to Center for Disease Control (CDC) guidelines. Two items asked participants about frequency of strenuous physical activity [31] and amount of sleep per night. Intake of fruits and vegetables, added sugar in foods and sugar-sweetened beverages [32] was assessed in three questions with categorical response options. Three additional items asked participants about their weight-related social support, self-efficacy and frequency of home food preparation.

#### 2.7.2. Workplace Outcomes

Six items with Likert scale or categorical response options asked participants to rate their current job performance [33], their energy to sustain work throughout the day, their job fulfillment, whether they would recommend their employer and their health-related absenteeism [33]. Items were selected to align with existing employer measures such as annual employee engagement survey metrics.

#### 2.7.3. Well-Being Outcomes

Three items were used to assess respondents’ emotional health, physical health and general personal goal attainment [34] and two items were used to assess overall life satisfaction. Response options used Likert scales and were again sourced from existing employer surveys where possible.

#### 2.7.4. Implementation Fidelity Measures

Eight items asked participants to report their satisfaction with program enrollment, the importance of the insurance incentive offered by their employer for participating, the desire for employer-offered health and well-being programs and their satisfaction with various program components (at 3 and 12 months only). These measures were tracked throughout the program alongside other fidelity measures using programmatic data.

#### 2.7.5. Demographics

Highest education status, race and ethnicity were collected as self-reported items from the survey, while gender and age were collected as self-reported data during the screening process.

#### 2.7.6. Insurance Claims Methods and Measures

Applicable healthcare utilization, prescription medication and disease diagnosis variables were collected from administrative health insurance claims and enrollment databases by a study team programmer for the year prior to the date of Profile enrollment for each participant. The same set of variables was also collected and summarized for the one year post-enrollment. Due to the COVID-19 pandemic disruptions in healthcare occurring at the end of this post-enrollment period, participants whose post-enrollment period spanned the state’s announced stay at home order date were excluded (*n* = 3). Prescription drug pharmacy claims were categorized by selecting specific American Hospital Formulary Service (AHFS) therapeutic classes thought to be impacted by weight loss [35]. The percent of participants who had a claim for any drug in a class is reported. Chronic care for asthma, type 2 diabetes, cardiovascular disease (CVD) and chronic obstructive pulmonary disease (COPD) was also reported using claims data. Disease categories from associated claims were derived from International Classification of Disease (ICD-10) codes [36] mapped to Johns Hopkins Expanded Diagnosis Clusters (EDCs) and/or Major Expanded Diagnosis Clusters (MEDCs) as appropriate [37]. Healthcare utilization data were estimated from claims, limited to one encounter per day for the following internally designated service categories: primary care, specialty care, outpatient, inpatient, emergency department and urgent care. Utilization categories were summarized into binary variables (i.e., those with any utilization for a given category over the time periods of interest).

#### 2.7.7. Profile Programmatic Data Methods and Measures

To measure additional program fidelity metrics, administrative programmatic data collected regularly by Profile were accessed by Profile staff and collated by the research team. Fidelity variables identified with programmatic data included the number of coaching sessions completed (total number of sessions, frequency of sessions, percent who completed the program target of 8 sessions in 3 months and percent who completed the insurance-incentive required 20 sessions), Profile meals purchased (number of transactions, number of items purchased and total dollar amount spent) and self-weights reported (number of weigh-ins, duration of time between first and last weigh-in and frequency of weigh-ins).

### 2.8. Analysis Plan and Sample Size

**Figure 1:**
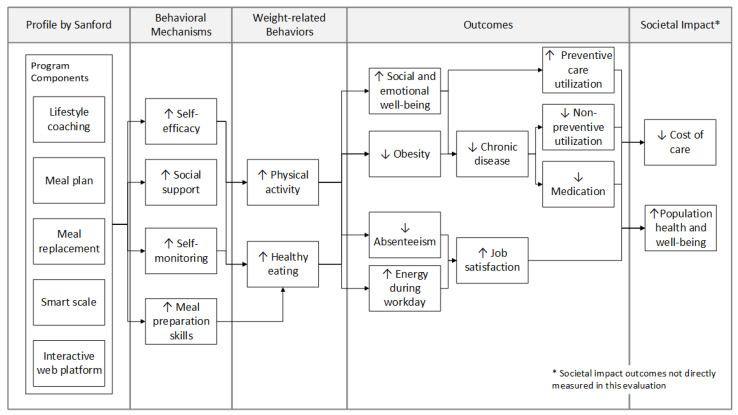
Conceptual model showing the hypothesized pathway from program components to behavioral mechanisms, behaviors and outcome measures in this study.

Figure 1 is a conceptual model showing the hypothesized pathway between each of the program components and measured outcomes. Descriptive statistics including frequencies, percentages, means and standard deviations were used to describe both self-reported and measured variables at baseline. To test the change in survey-measured outcomes from enrollment to 3 and 12 months post-enrollment, individual paired t-tests, Wilcoxon sign-rank tests and binomial test of proportions were conducted with continuous, categorical and dichotomous variables, respectively. Binomial tests for proportions were also used to test the pre/post change in dichotomous claims-measured outcomes. Primary analyses were conducted with an a priori alpha of 0.05. However, additional analyses were adjusted for multiple comparisons using a Bonferroni adjustment (α/m number of tests). Descriptive statistics were used to assess fidelity of implementation (Aim 3) over the course of one year post-enrollment.

Profile was offered to all health system employees and covered adult dependents, estimated to total 30,000 individuals. Based on Profile enrollment in other settings, it was estimated that 2–3% of the eligible population (*n* = 800) would enroll in Profile, 35–40% of whom (*n* = 300) were likely to complete the evaluation study enrollment. An estimated 75% (*n* = 225) would complete 12-month study follow-up. Based on organization data and 2016 Minnesota Behavioral Risk Factor Surveillance System (BRFSS) data [38], the expected average BMI for the study population was 27.84 kg/m^2^ with standard deviation of 2.54 kg/m^2^. After setting the study enrollment goal of 300, there was lower than expected Profile program enrollment, likely from lack of brand recognition and/or competing program options, which diminished the pool of study-eligible individuals. To address this, a post hoc power analysis was done to reassess sample size needs. Preliminary data from 53 respondents were used to understand BMI categories in target population. With 150 participants and an estimated 25% loss to follow-up the study has 80% power to detect a clinically meaningful 5% reduction in BMI using a two-tailed paired t-test with an alpha of 0.05.

## 3. Results

### 3.1. Population Description

**Figure 2:**
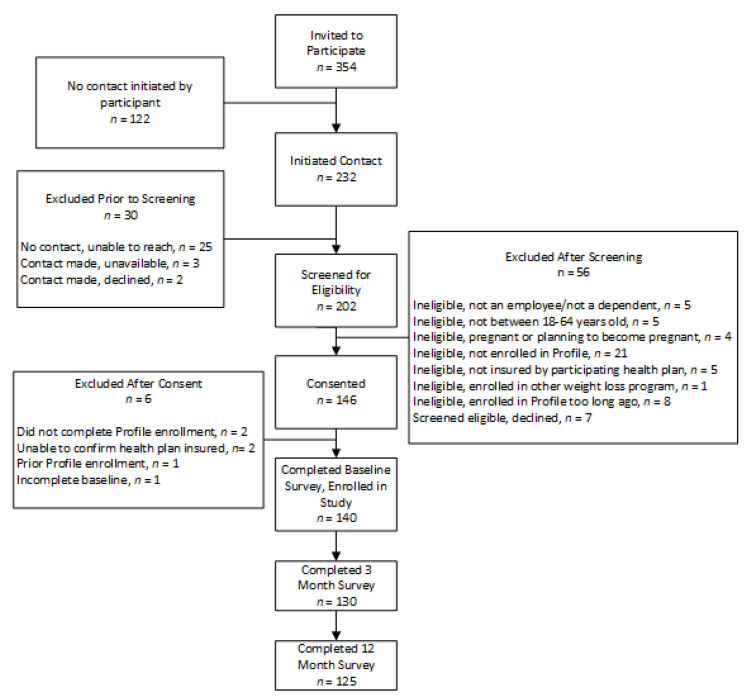
Study flow from program enrollment through study recruitment and data collection.

In total, 354 employees were documented as enrolling in Profile and invited to participate in the current evaluation. Sixty-six percent (*n* = 232) were interested in participating and initiated contact and 87% (*n* = 202) of these were screened for eligibility. In total, 56 were excluded after screening; 49 of those were ineligible and 7 declined post-screening. Of the 146 who were eligible and consented, 96% (*n* = 140) completed the baseline measures and were enrolled in the study. Overall, the recruitment rate was 40% of those originally invited to participate. The average number of days between program enrollment and baseline survey completion was 11.6 (SD = 12.0) days. Ninety-three percent (*n* = 130) of those enrolled completed the 3-month survey and 89% (*n* = 125) completed the 12-month survey. Figure 2 presents some additional details.

Baseline survey results are presented in Table 1. The majority of study participants (*n* = 140) were non-Hispanic, White and female, with an average age of 51.2 ± 9.8 years and 17.9% had a graduate degree. Mean participant-reported BMI from surveys was 33.2 ± 6.5 kg/m^2^, with 25.0% categorized as having overweight and 64.7% as having obesity. Approximately one fifth had tried losing weight 10 or more times previously and the self-reported ideal weight loss goal was 22.0%. Ten percent of participants reported doing strenuous physical activity five or more times per week and 20.0% reported consuming five or more servings of fruits and vegetables per day. Most (64.0%) reported generally avoiding sugar-sweetened beverages, but few (9.4%) generally avoided added sugars. While weight-related social support was high (79.3%), 18.6% reported high weight loss-related self-efficacy and few (17.9%) reported preparing 10 or more meals a week at home. Twenty-seven percent of participants reported at least some health-related absenteeism and about half (42.9%) rated their job performance at a 9 or above on a scale of 0–10. Approximately a quarter (23.0%) rated their job fulfillment as a 9 or above on the same scale and even fewer reported having the energy to sustain a workday (19.3%). Some participants reported that emotional and physical health concerns do “not at all” impact their lives (35.0% and 30.7%, respectively). On a scale of 0–10, life satisfaction of 9 or higher was reported by 17.6% of participants.

### 3.2. Survey-Measured Outcomes

**Figure 3:**
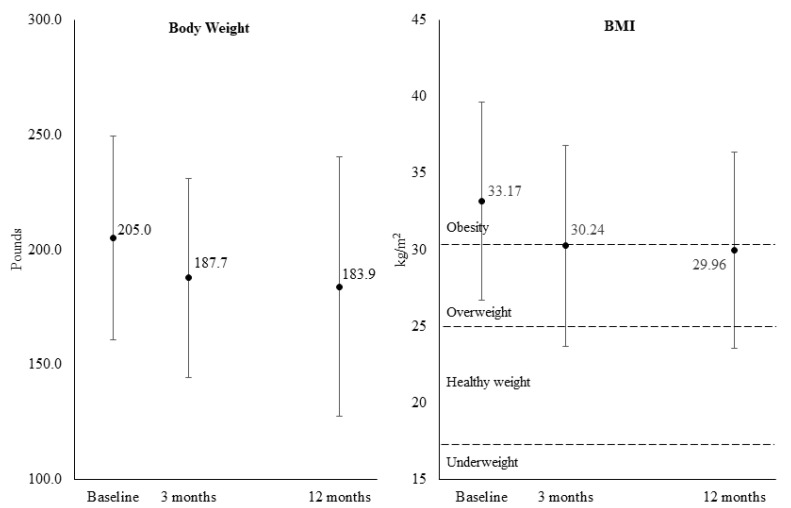
Average body weight and body mass index (BMI) of participants at baseline, 3 months and 12 months. Note: Graphs represent all available data at each time point and are not paired across time.

Full results can be found in Table 2. There was a statistically significant and clinically meaningful reduction in participant-reported BMI from baseline to 3 months (∆M = −2.72, SD = 1.79 kg/m^2^, *p* < *0*.0001). This reduction was even larger at 12 months but had higher variability (∆M = −3.94, SD = 6.51 kg/m^2^, *p* < *0*.0001). Figure 3 shows cross-sectionally the average weight and BMI at each time point. On average, participants lost 17.1 lbs at 3 months (SD = 11.52) and 17.5 lbs at 12 months (SD = 24.08). Seventy-three percent of participants lost a clinically meaningful (5% or more) amount of weight at 3 months and 56% at 12 months. Slightly more lost weight at 3 and 12 months, (83% and 65%, respectively), if the threshold is lowered to 3% body weight lost—a meaningful percentage for community weight loss programs [39]. Significant weight change from baseline was observed at both 3 and 12 months (3-month M = −8.12%, SD = 8.2%; 12-month M = −8.15%, SD = 10.54%). There were statistically significant and maintained self-reported changes in all measures of dietary intake (increased fruit and vegetables, reduced sugar-sweetened beverages and reduced added sugar) but not physical activity at 3 and 12 months. Improvements were seen in some behavioral mechanisms of weight-related behavior change (i.e., home food preparation) but not for weight-related social support or self-efficacy. Statistically significant improvements were seen in emotion and physical health concerns getting in the way of life, and these were maintained at 12 months. The same was not seen for other measures of well-being (i.e., goal attainment and thinking about good things that happen), however overall life satisfaction was higher at both 3 and 12 months compared to baseline. There was a small but statistically significant increase in absenteeism at 12 months, but not at 3 months, and the statistical significance did not hold after adjusting for multiple comparisons. There were improvements in energy throughout the workday at both 3 and 12 months but not in job performance or job fulfillment (with the exception of borderline improvements in reported job performance at 3 months, *p* = 0.042, which did not hold after adjusting for multiple comparisons).

### 3.3. Claims-Measured Outcomes

In the year pre-enrollment, 87% of included participants had at least one primary care encounter. Urgent care, emergency department outpatient and inpatient utilization was lower (ranging from 5% to 34% with at least some utilization in these categories). There were no differences in healthcare utilization outcomes in the year post-enrollment. The most common claims-measured disease outcome pre-enrollment was cardiovascular disease (44.0%). Fewer participants had a claim in the 12-month pre period related to asthma or type 2 diabetes (12.0% and 4.8%, respectively.) There were no differences in the proportion of people with these conditions pre and post enrollment. The most commonly prescribed medications were respiratory (33.6%), followed by gastroenterological (30.4%) and pain medications (26.4%). There were no statistically significant differences in medications pre and post enrollment. Table 3 presents some additional details.

### 3.4. Profile Implementation Fidelity

Program satisfaction was high at 12 months with 82% being somewhat or very satisfied (42% being very satisfied), which was slightly lower than overall satisfaction at 3 months (91% somewhat or very satisfied, data not shown). Likelihood to recommend the program was also high at 12 months (M = 6.88, SD = 2.92 on a scale of 0 = not at all likely to 10 = extremely likely). At baseline, participants reported high satisfaction with program enrollment (64% said “yes, definitely” satisfied), 42% said it was somewhat or very important that the program was offered as a way to get an insurance incentive and 66% strongly agreed that they desired future health and well-being programs from their employer. Full implementation fidelity results can be found in Table 4.

Utilization of program foods, which were not required but highly encouraged, was high with the average participant making 22 unique food purchases and purchasing on average 133 items over 12 months, but variability in these measures was also high. The average amount spent on purchases was $2348 (SD = $2352) over the year and the majority of participants reported using program foods at both 3 and 12 months (90% and 76%, respectively). However, satisfaction with the food at 12 months was modest (31% very satisfied) falling from 46% at 3 months (data not shown). Participants completed an average of 18 coaching sessions in 12 months, with 69% completing 8 sessions in the first 3 months but only 39% completing 20 sessions in 12 months. Participants recorded an average of 75 weights (SD = 76 weights) over 12 months, with the average duration between first and last recorded weight being 263 days (SD = 111 days) or approximately 9 months. The average number of weights per month was 7.8 (SD = 5.8) with an average of 6.4 (SD = 7.6) days between weights. Almost all participants reported still meeting with a coach at 3 months, which fell to 63% at 12 months. Of those still meeting with a coach at 12 months, general satisfaction was high (M = 8.2, SD = 2.8 on a scale of 0 = worst coaching possible to 10 = best coaching possible). Confidence in weight management and lifestyle behavior following coaching was more modest (41% and 52% very confident, respectively).

## 4. Discussion

Profile is a theory-informed, science-driven weight management program designed in close partnership with a healthcare system (Sanford Health). This article presents the fidelity and outcomes of a pragmatic, multi-dimensional evaluation of Profile, a novel healthcare-designed behavioral weight management program that utilizes phone coaching, self-monitoring and meal replacements delivered in a worksite setting. The findings from this evaluation establish the fidelity with which this program was implemented when offered as a well-being incentive to a separate healthcare employee population by their employer and insurer. The fidelity of implementation and satisfaction were high, with some concrete opportunities for improvement. The evaluation also provides preliminary evidence of the effectiveness in this population with self-reported improvements in many key outcomes (e.g., BMI, body weight and dietary measures). Although self-reported height and weight have been shown to be impacted by self-presentation bias, these measures are generally accepted as appropriate for comparing longitudinal changes over time as they have very strong correlation with objectively measured height and weight [25,40,41]. Lastly, there was no strong evidence of improvement in other claims-measured outcomes.

Data reported here show that a high percentage of eligible participants were recruited, enrolled and retained in the study over 12 months. This type of frame data and ability to report true response rates are not often available in this type of evaluation, which provides greater confidence in the results. The high recruitment and retention rate also increases the likelihood that the study population is representative of the target population (i.e., those who participated in Profile). This evaluation did not examine the reach of *program* enrollment and researchers have consistently documented a higher proportion of women participating in behavioral weight management programs [42] such as Profile. The same pattern is likely true here with almost 90% of participants being female. Interestingly, the makeup of the study population was similar to the larger employee population in terms of other demographic characteristics, according to unpublished internal employer data and other worksite weight management programs [43].

Program fidelity and satisfaction were examined as a part of this evaluation and showed high adherence across the three major components: coaching sessions, program food purchases and self-weighing. There was lower engagement with these components than has been shown in published evaluations of similar weight loss programs [44,45]. That said, there was high variability in participation and participation waned slightly by 12 months. Approximately 70% of participants completed the program goal of 8 coaching sessions in 3 months and only about 40% completed the 20 sessions required for the insurance well-being incentive. However, the average participant still completed 18 sessions, 75 self-weights and 22 purchases over 12 months. Notably, the average amount spent on program food was over $2000, suggesting a large proportion of participants’ annual spending on food (estimated as approximately $6500) was spent on program food [46,47]. It is possible that cost of the program was a barrier for some in this employed population and may be a barrier for accessibility in underserved communities. However, the cost of the program and Profile foods is substantially less than the cost of other obesity treatment options such as registered dietician appointments in the clinical setting or weight loss medications [48]. Exploring efforts to increase the insurance coverage of evidence-based behavioral weight management programs such as Profile would increase the affordability and access for more populations. As with participation, program satisfaction was also high with slight declines over time. This was particularly true for overall program satisfaction, which fell from 63% at 3 months to 42% at 12 months. Although satisfaction in coaching increased slightly over time, this is likely due to the fact that only those who continued with coaching by 12 months were asked about satisfaction, suggesting those who were unsatisfied likely did not continue with this component. Among the 76% who continued to purchase program food at 12 months (which fell from 90% at 3 months), satisfaction with this component also declined slightly and was lower overall compared to other components.

These variable participation and satisfaction results notwithstanding, self-reported weight loss was substantial with the average participant losing over 8% of their body weight (average weight loss was 17.5 pounds at 12 months) and almost three BMI points at 3 months and maintaining or slightly increasing this weight change at 12 months. Although these findings are limited by self-reported weight data and lack comparison to a randomized comparison group, the high response rate at 12 months and the longitudinal design increase confidence in the findings. Further, the documented upward trend in self-reported obesity across the country and in Minnesota provides additional confidence in the results from this study [25]. Similarly, self-reported dietary intake and home food preparation improved at both 3 and 12 months compared to baseline. The same was not seen for reported physical activity or weight-related social support or self-efficacy. It is possible that the lack of findings in this area, as well as the lack of significant weight loss after 3 months, could be due to the incorrect identification of the true mechanisms of the intervention. Future work could use intervention mapping strategies to identify and test mechanisms contributing to initial and continued weight loss beyond 3 months [49].

These pilot results should be used to conduct a future full-scale evaluation with a randomized design and gold-standard objective measurement to account for potential confounding or bias. Nearly three-fourths of participants lost a clinically meaningful 5% of body weight at 3 months and over half maintained 5% or greater weight loss at 12 months, results which are in line with similar clinically oriented weight management programs [9,11]. Eighty-three percent of participants reached the threshold of 3% body weight loss at 3 months, which is a threshold criterion for effective community-based interventions [39], with 65% maintaining that loss at 12 months. Although these may be relatively small decreases in weight, without intervention weight tends to increase over time [25], and even short-term weight change can have positive health and well-being impacts [50].

In fact, important corresponding improvements were seen in the impact emotional and physical health concerns had on program participants’ lives at both 3 and 12 months. Overall life satisfaction increased over a half a point (on an 11-point scale) as well. These are important changes to note, as baseline well-being and emotional health was lower in this population compared to other employed adults in this region covered by the same health plan [51]. Claims-measured health outcomes such as chronic disease diagnoses, medications and utilization of non-preventive services were not lower in the year following enrollment. However, the prevalence of claims-measured chronic conditions at baseline were modest but in alignment with other populations [52,53]. It is possible that program-induced lifestyle changes did not result in a large enough weight change to see short-term changes in these measures as compared with more extreme weight loss efforts such as weight loss medication or surgery [54]. It is also possible that these metrics may have shown subsequent improvement following weight loss rather than in parallel with weight loss (e.g., change from 12 to 24 months post-program enrollment rather than from 0 to 12 months post-enrollment).

Lastly, this evaluation sought to understand this type of program’s impact on workplace productivity. Although health-related absenteeism was slightly worse at 12 months, this did not hold following adjustment for multiple comparisons, and participant-reported energy to sustain a full workday improved dramatically at 3 and 12 months (a half point increase on a four-point scale). Similar improvements were not seen in reported job performance or job fulfillment, which were relatively high at baseline. This is the first published evaluation showing improved metrics of job performance following an insurance-offered weight loss program in a healthcare employee population [55]. Lastly, of importance to employers and insurers, participants felt strongly that future well-being programs such as this should be offered by their employers and offering this program as a well-being insurance incentive was at least modestly important.

This evaluation presented here highlights a number of key strengths and some limitations. Although the study population is relatively representative of the employer population, there is limited variability in the diversity of program participants. The evaluation was able to recruit a high percent of program participants but highlights the need to design and implement culturally sensitive engagement approaches. Similarly, employees who participated in Profile and enrolled in this study were highly motivated with significant prior weight loss attempts. Although this may limit the generalizability of this study, a recent review estimates 42% of the population has tried to lose weight, suggesting weight loss motivation is high in the general population as well [56]. Further exploration is needed to understand whether this type of weight loss program can be effective for those with fewer or no previous weight loss attempts. A noted strength of this approach was the use of existing data sources to evaluate this program, minimizing participant burden and cost. However, the study design did not include a control group or randomization so we cannot say whether changes in weight or other outcomes were attributable solely to the program; however, the use of a longitudinal, pre/post design provides inherent control of potential person-level confounders that do not change over time, such as demographics, without the need for a separate comparison group. Due to the study’s pragmatic design, we were not able to use gold-standard diet and physical activity measures such as dietary recall and accelerometry. However, the measures we used were standard, consistent over time and correlated with gold-standard measures. This, in combination with the fact that we saw changes in the three key dietary measures included as well as change in BMI and body weight, give us confidence in our results. The exclusion of more onerous diet and physical activity measures does not negate the current findings, although it does limit what can be said about the program’s impact on caloric intake or expenditure, specifically. A key strength of this evaluation was the high participation rate and the fact that the study was offered to all of the organization’s enrolled Profile members. However, there is still the possibility for selection bias among those who chose to participate in this study. While it is possible that the insurance incentive led to bias in participation rates compared to other non-incentivized weight loss programs, this employer has had consistent engagement in health insurance incentive completion across roles, incomes and health conditions, thus there is no reason to assume that those who complete the incentive are different from those who do not.

Study participation could also have had an independent impact on outcomes. Other key strengths included the gathering of participant voice via surveys and the triangulation of multiple data sources to efficiently evaluate outcomes of interest to diverse stakeholders (e.g., employees, employers, program developers and health systems). Based on the findings of high implementation fidelity, significant and clinically meaningful weight loss and many positive changes in behaviors and well-being outcomes over time, this study found that Profile is a promising behavioral weight loss program when offered to employees in a healthcare setting.

## 5. Conclusions

The results from this pragmatic evaluation show that the Profile weight management program was implemented with high fidelity and participant satisfaction. Improvements in many key outcomes were seen, with an approximate 8% loss in average body weight at 3 months, improvements in life satisfaction and energy to sustain a full workday and reductions in emotional and physical health concerns; however, there was no impact on claims-related outcomes. The results presented here are a first step in establishing the evidence base for this program and providing information for powering and implementing a full-scale randomized trial.

## Figures and Tables

**Table 1:** Baseline characteristics of participants, *n* = 140 ^a^.

Baseline Characteristics	M (SD) or % (*n*)
**Demographics**	
Race	
White	93.6% (131)
American Indian/Alaska Native	0.0% (0)
Native Hawaiian/other Pacific Islander	0.0% (0)
Other race	5.7% (8)
Ethnicity	
Non-Hispanic	98.6% (138)
Gender	
Female	89.3% (125)
Age, years	51.2 (9.8)
Education	
8th grade or less	0.0% (0)
Some high school	0.0% (0)
High school diploma/GED	5.7% (8)
Technical training/associate degree	13.6% (19)
Some college	12.1% (17)
College degree	50.7% (71)
Graduate studies	17.9% (25)
**Weight-related outcomes**	
Self-report weight, pounds	205.0 (44.3)
Self-report body mass index (BMI), kg/m^2^	33.2 (6.5)
Weight status	
Underweight: BMI < 18.5	0.0% (0)
Normal: BMI 18.5–24.9	10.3% (14)
Overweight: BMI 25.0–29.9	25.0% (34)
Obese: BMI 30+	64.7% (88)
Prior weight loss, 10 or more times	20.7% (29)
Ideal weight loss, % of baseline weight	22.0 (9.3)
**Weight-related behaviors**	
Strenuous physical activity, 5+ times vigorous activity a week	10.0% (14)
Fruit and vegetable intake, 5+ servings/day	20.0% (28)
Sugar-sweetened beverage intake, generally avoid these drinks	64.0% (89)
Added sugar intake, generally avoid these foods	9.4% (13)
**Weight-related behavioral mechanisms**	
Prepare meals at home, more than 10 meals	17.9% (25)
Weight-related social support, yes	79.3% (111)
Weight loss related self-efficacy, extremely certain	18.6% (26)
**Well-being outcomes**	
Emotional health concerns impact life, not at all	35.0% (49)
Physical health concerns impact life, not at all	30.7% (43)
Goal attainment, strongly agree	30.0% (42)
Think about good things that happen, often	56.8% (79)
Overall life satisfaction ^b^, 9 or above	17.6% (24)
**Workplace productivity outcomes**	
Health-related absenteeism, full or partial day, at least one partial day	27.1% (38)
Job performance ^b^, 9 or above	42.9% (60)
Energy to sustain, strongly agree	19.3% (27)
Job fulfillment ^b^, 9 or above	23.0% (32)

^a^ Total denominator for each item varies slightly depending on item response rates. ^b^ Defined as 9 or 10 on a 0–10 scale.

**Table 2:** Change in participant’s survey-measured weight-related, workplace and well-being outcomes at 3 and 12 months.

Outcomes	Baseline-3 Months, *n* = 130 ^a^		Baseline-12 Months, *n* = 125 ^a^	
	∆M (SD) ^b^	*p*-Value ^c^	∆M (SD) ^b^	*p*-Value ^c^
Weight-related outcomes				
Self-report BMI, kg/m	−2.72 (1.79)	<0.0001	−3.94 (6.51)	<0.0001
Body weight lost, lbs	17.08 (11.52)	<0.0001	17.50 (24.08)	<0.0001
Percent body weight lost, % lbs lost	8.12 (5.00)	<0.0001	8.16 (10.54)	<0.0001
Weight-related behaviors				
Strenuous physical activity, number of times/week	−0.50 (13.94)	0.6891	−0.45 (14.44)	0.7345
Fruit and vegetable intake, number of servings/day	1.61 (1.80)	<0.0001	1.11 (1.82)	<0.0001
Sugar-sweetened beverage intake, number of servings/day	−0.52 (1.10)	<0.0001	−0.39 (0.92)	<0.0001
Added sugar intake, number of servings/day	−1.98 (1.52)	<0.0001	−1.48 (1.67)	<0.0001
Weight-related behavioral mechanisms				
Prepare meals at home, number of meals/week	2.72 (12.24)	0.0136	1.75 (4.64)	<0.0001
Weight-related social support, yes	0.05 (0.43)	0.2281	0.03 (0.41)	0.5034
Weight loss related self-efficacy, 4 point Likert scale	0.03 (1.13)	0.5659	n/a	n/a
Well-being outcomes				
Emotional health concerns impact life, 4 point Likert scale	−0.25 (0.77)	0.0002	−0.17 (0.92)	0.0174
Physical health concerns impact life, 4 point Likert scale	−0.35 (0.99)	<0.0001	−0.25 (0.96)	0.0007
Goal attainment, 4 point Likert scale	−0.06 (1.23)	0.6355	−0.08 (1.23)	0.4042
Positive thinking, 4 point Likert scale	0.08 (0.48)	0.0669	0.09 (0.55)	0.0699
Overall life satisfaction, 11 point scale	0.53 (1.23)	<0.0001	0.52 (1.65)	0.0008
Workplace productivity outcomes				
Health-related absenteeism, partial or full day in prior month	0.06 (0.42)	0.1451	0.10 (0.45)	0.0180
Job performance, 11 point scale	0.21 (1.17)	0.0419	0.11 (1.14)	0.2701
Energy to sustain, 4 point Likert scale	0.58 (1.15)	<0.0001	0.42 (1.31)	0.0002
Job fulfillment, 11 point scale	0.15 (1.50)	0.2620	0.26 (1.70)	0.0972

^a^ Total denominator for each item varies slightly depending on item response rates. ^b^ *p*-values for ordinal categorical variables are derived from tests of median values, however for descriptive purpose means and standard deviations are displayed. ^c^ The results from paired t-tests, Wilcoxon sign rank tests or binomial test of proportions depending on outcome variable type (continuous, ordinal or dichotomous, respectively). n/a = not applicable.

**Table 3:** Change in participant’s claims-measured healthcare outcomes from 12 months pre- to 12 months post-program enrollment, *n* = 125 ^a,b^.

Outcomes	Pre	Post	*p*-Value ^c^
	% (*n*)	% (*n*)	
Healthcare utilization outcomes			
Primary care Encounters, at least one	87.2% (109)	86.4% (108)	0.8518
Outpatient encounters, at least one	34.4% (43)	31.2% (39)	0.5900
Inpatient encounters, at least one	4.8% (6)	7.2% (9)	0.4243
Emergency department encounters, at least one	16.0% (20)	16.8% (21)	0.8644
Urgent care encounters, at least one	26.4% (33)	28.8% (36)	0.6712
Chronic disease outcomes			
Asthma	12.0% (15)	16.0% (20)	0.3621
Cardiovascular disease	44.0% (55)	51.2% (64)	0.2544
Type 2 diabetes	4.8% (6)	3.2% (4)	0.5270
Medication outcomes			
Anti-diabetic prescription, at least one	6.4% (8)	7.2% (9)	0.8016
Cardiovascular prescription, at least one	19.2% (24)	18.4% (23)	0.8714
Gastroenterological prescription, at least one	30.4% (38)	21.6% (27)	0.1127
Hypolipidemic prescription, at least one	13.6% (17)	18.4% (23)	0.3006
Pain prescription, at least one	26.4% (33)	24.8% (31)	0.7719
Psychotropic prescription, at least one	6.4% (8)	7.2% (9)	0.8016
Respiratory prescription, at least one	33.6% (42)	34.4% (43)	0.8938

^a^ Excludes *n* = 3 participants whose 12-month post period ended after 28 March 2019 (the start of Minnesota’s COVID-19 stay at home order). ^b^ Excludes *n* = 10 participants without continual insurance coverage by the included health plan during the full 24-month period. ^c^ *p*-values from test of proportions.

**Table 4:** Satisfaction and fidelity of program implementation from survey or administrative program data, *n* = 140 ^a^.

Fidelity Measures	*n* (%) or M (SD)
General program	
Satisfaction with program at 12 months, very satisfied	41.8% (51)
Likelihood to recommend program at 12 months, scale of 0 to 10	6.88 (2.92)
Program enrollment	
Satisfaction with enrollment at baseline, yes, definitely	64.0% (87)
Importance of insurance incentive at baseline, somewhat or very important	41.8% (56)
Desire for future health/well-being programs from employer, strongly agree	66.2% (90)
Program food purchases ^b^	
Purchases over 12 months, *n*	21.72 (16.44)
Items purchased over 12 months, *n*	132.67 (111.05)
Total amount spent over 12 months, dollars	$2348 ($2352)
Percent who never used program foods	8.8% (11)
Percent using program foods at 3 months	89.8% (114)
Percent using program foods at 12 months	76.0% (92)
Satisfaction with food at 12 months, very satisfied	30.8% (28)
Program self-weights	
Self-weights completed over 12 months, *n*	74.67 (76.26)
Duration of self-weight from first to last over 12 months, days	263.34 (110.68)
Frequency of self-weights, weights per month	7.80 (5.81)
Frequency of self-weights, days between weights	6.43 (7.62)
Program coaching	
Sessions completed in 12 months, *n*	18.03 (12.20)
Percent who did 8 sessions in 3 months	69.3% (95)
Percent who did 20 sessions in 12 months	38.7% (53)
Percent meeting with a coach at 3 months	95.2% (120)
Percent meeting with a coach at 12 months	63.1% (77)
Satisfaction with coach at 12 months, scale of 0 to 10	8.19 (1.79)
Weight management confidence following coaching at 12 months, very confident	40.8% (31)
Lifestyle behavior confidence following coaching at 12 months, very confident	52.0% (39)

^a^ The denominator varies slightly based on measurement time point (baseline, 3 or 12 months), data collection method (survey vs. administrative program data) and individual item response. ^b^ Excludes *n* = 10 participants who had multiple program participants in a single household.

## Data Availability

The data presented in this study are available on request from the corresponding author. The data are not publicly available due to privacy reasons.

## Supplementary Materials

The following are available online at https://www.mdpi.com/article/10.3390/ijerph18115901/s1. Supplementary Material: Profile Experience Baseline Survey.
