# Supplementary material for: Pragmatic Evaluation of a Health System-Based Employee Weight Management Program
Source: Int J Environ Res Public Health. 2021 May 31;18(11):5901. doi: 10.3390/ijerph18115901 (PMC8199381; doi:10.3390/ijerph18115901)
Supplement: Supplementary file 1 [file ijerph-18-05901-s001.zip › ijerph-1210541-supplementary.pdf]

International Journal of
Environmental Research and Public Health

MDPI

Supplementary Material: Profile Experience Baseline Survey

The following questions are about your experience with Profile. Answer as honestly as you can; there are no right or wrong answers. If you feel uncomfortable or do not wish to answer any particular questions, you may skip those.

1. Where did you hear about Profile? Please select all that apply.
Family member or spouse
Coworker
Email
Presentation
Other
Other, please specify:___________________

2. Before you enrolled in Profile, were you given all the information that you needed or wanted about the program?
Yes, definitely
Yes, somewhat
No

3. Why did you choose to participate in Profile? (Check all that apply).
I wanted to get the reward offered by my employer
I wanted to better understand my health
I wanted to take advantage of the available well-being tools, resources, and activities.
I wanted to improve my health and well-being
I wanted help supporting a lifestyle change I am already making

4. Individuals that complete Profile qualify for the [Organization] BeWell incentive. How important was this in your decision to enroll in Profile?
Very important
Somewhat important
Somewhat not important
Not at all important

Please state the extent to which you agree or disagree with the following statement.

5. I would like my employer to offer health and well-being programs like Profile in the future.
Strongly disagree
Somewhat disagree
Neither agree nor disagree
Somewhat agree
Strongly agree

The following questions are about your general health and well-being.

6. How satisfied are you with your life? Please answer on a scale of 0-10, where 0 is not at all satisfied and 10 is extremely satisfied.
0, Not at all satisfied
1
2
3
4
5
6
7
8
9
10, Extremely satisfied

7. How often do you take time to think about the good things that happen to you?
Often
Sometimes
Rarely
Never

8. In general, how enjoyable and fulfilling is your main job or daily work? Please answer on a scale of 0-10, where 0 is not at all enjoyable and fulfilling and 10 is extremely enjoyable and fulfilling.
0, Not at all enjoyable and fulfilling
1
2
3
4
5
6
7
8
9
10, Extremely enjoyable and fulfilling

Now please think of your work experiences over the past 4 weeks (28 days). We'd like to know how many days you spent in each of the following work situations. In the past 4 weeks (28 days), how many days did you...

9. Miss an entire work day because of problems with your physical or mental health? (Please include only days missed for your own health, not someone else's health.)
____ days (0-28)

10. Miss part of a work day because of problems with your physical or mental health? (Please include only days missed for your own health, not someone else's health.)
____ days (0-28)

11. On a scale from 0 to 10 where 0 is the worst job performance anyone could have at your job and 10 is the performance of a top worker, how would you rate your overall job performance on the days you worked during the past 4 weeks (28 days)?
0, Worst performance
1
2
3
4
5
6
7
8
9
10, Top performance

To what extent do you agree or disagree with the following statements?

12. I am able to sustain the level of energy I need throughout the workday
Strongly disagree
Somewhat disagree
Neither agree nor disagree
Somewhat agree
Strongly agree

12a. [for employees only – not dependents] I would recommend [Organization] to others as a good place to work
Strongly disagree
Somewhat disagree
Neither agree nor disagree
Somewhat agree
Strongly agree

13. How much do emotional health concerns, such as feeling depressed or anxious, get in the way of your life?
Not at all
A little bit
Some
Quite a bit
Completely

14. How much do physical health concerns get in the way of your life?
Not at all
A little bit
Some
Quite a bit
Completely

15. To what extent do you agree or disagree with the following statement? I will be able to achieve most of the goals that I have set for myself.
Strongly disagree
Somewhat disagree
Neither agree nor disagree
Somewhat agree
Strongly agree

When you answer the following questions, please think about your behaviors before you enrolled in Profile.

16. How many servings of fruits and vegetables do you eat each day? A serving is 1 medium fruit, 1 cup fresh greens, 1 cup raw vegetables, 1/2 cup fruit or cooked vegetables, or 4 oz. 100% fruit juice.
0
1
2
3
4
5
6
7
8
9
10
11 or more

17. How many sugar-sweetened drinks do you normally drink per day? Count things such as soft drinks, fruit drinks, and sweetened coffee or cocoa drinks, but do not include diet drinks.
I generally avoid these drinks
1
2
3
4
5
More than 5 per day

18. How many foods with added sugar do you normally eat per day? Count foods such as candy, cookies, and desserts.
I generally avoid these foods
1
2
3
4
5
More than 5 per day

19. In the past 7 days, how many meals have you prepared in your own kitchen? Consider both your afternoon and evening meals.
__ meals (enter a number between 0 and 14)

20. Do you ever eat large amounts of food and feel like you can't stop or control your eating?
Yes
No

If you have concerns about your eating, talk to your doctor or contact Melrose Center at 952-993-6200.

21. Considering a 7-day period (a week), how many times on average do you do *strenuous* exercise (where your heart beats rapidly) for more than 15 minutes during your free time?
____ times per week

22. How many hours of sleep do you get each day?
__ hours (enter a number between 0 and 24)

23. When you need advice or support, is there someone you can turn to?
Yes
No
I don't know

24. Besides your Profile coach, when you need advice or support with your weight loss goals, is there someone you can turn to?
Yes
No
I don't know

25. How many times in the past have you seriously tried to lose weight?
0 times
1-2 times
3-5 times
6-10 times
10 or more times

26. How certain are you that you will stay committed to a weight loss program for the time it will take you to reach your goal?
Not at all certain
Slightly certain
Somewhat certain
Quite certain
Extremely certain

27. Is there anything else you'd like to share about your experience with Profile? (open-ended)

The following questions will help us understand your responses better.

28. Do you consider yourself to be Hispanic or Latino?
No, not Hispanic/Latino
Yes, Hispanic/Latino

29. Which of the following do you consider yourself? (Mark all that apply.)
Asian
Black or African American
White
American Indian or Alaska Native
Native Hawaiian or other Pacific Islander
Other, please specify: _____________

30. How much schooling have you had?
8th grade or less
Some high school
High school diploma or GED
Technical training or Associate degree
Some college
College degree
Graduate studies

31. About how tall are you without shoes?
__ feet
__ inches

32. About how much do you weigh without shoes?
__ pounds

33. What is the ideal weight you would like to reach by the end of the Profile program?
__ pounds

Thank you for taking the time to complete this survey. Your answers are very important. We will contact you again in about 3 months for the next survey.
